# Prevalence of tobacco use and nicotine dependence among medical interns in Nepal: A cross-sectional study

**DOI:** 10.1371/journal.pone.0359298

**Published:** 2026-10-21

**Authors:** Krishna Subedi, Gitu Gaire, Bhawana Karki, Sandeep Neupane

**Affiliations:** 1 Department of Community Dentistry, Gandaki Medical College Teaching Hospital and Research Centre, Pokhara, Nepal; 2 Department of Internal Medicine, Institute of Medicine, Kathmandu, Nepal; Federal University of Paraiba, BRAZIL

## Abstract

This study was conducted to assess the prevalence of tobacco consumption, nicotine dependence and factors associated with tobacco use among medical interns in Nepal. Data was collected from February to April 2023 using an online questionnaire distributed through multiple platforms, with weekly reminders for five weeks. The questionnaire included demographic characteristics, tobacco use status, type of tobacco, age at initiation, and the six-item Fagerström Test for Nicotine Dependence. A minimum sample of 324 participants was required, and 389 participants were targeted to allow for potential non-response. Of the 798 interns who received the questionnaire, 381 completed it, for a response rate of 47.74%. The majority of participants were male, 54.33% (n = 207) and studying Bachelor of Medicine and Bachelor of Surgery, 64.57%(n = 246). The prevalence of tobacco use was 18.11% (n = 69), including 13.12% smokers (n = 50) and 4.99% smokeless tobacco users (n = 19). Smoking was significantly more common among male and MBBS interns. Exploratory, post-hoc multivariable logistic regression showed that female interns had lower odds of tobacco use than male interns (OR = 0.18; 95% CI: 0.09–0.36). Among the 50 smokers, one participant did not complete the nicotine dependence questionnaire; therefore, 49 smokers were included in the nicotine dependence analysis. Among smokers, nicotine dependence was low in 73.47% (n = 36), moderate in 24.49% (n = 12), and high in 2.04%(n = 1). Among the tobacco users, 44.93%(n = 31) responded that the reason for the initiation of tobacco products was self-interest, followed by peer pressure, 23.19%(n = 16). Tobacco use was less common among medical interns than in the general population, likely reflecting their higher educational attainment. Among those who smoked, most exhibited low levels of nicotine dependence. Self-interest and peer pressure were the primary reasons for tobacco initiation. The exploratory associations should be interpreted cautiously and considered hypothesis-generating.

## Introduction

Tobacco use is the leading cause of cardiovascular disease [1], cancers [2], respiratory disease [3], premature births, low birth weight infants [4], and periodontal diseases [5]. Tobacco consumption is one of the major public health concerns worldwide, with high morbidity and mortality [6], which is the single most preventable cause of death [7]. Tobacco products contain a variety of toxic substances, including nicotine, which is the most addictive component [8]. Nicotine addiction remains one of the easiest addictions to fall into and can develop rapidly into one of the toughest addictions to get rid of [9].

According to the World Health Organization (WHO), about 1.3 billion people worldwide consume some form of tobacco products, and 80% of those belong to low and middle-income countries [10]. In Nepal, 28.9% of adults aged 15–69 (48.3% of men and 11.6% of women) were current tobacco users in any form, amounting to 3.8 million adults [11].

Medical professionals are at the center for controlling tobacco use among patients. Furthermore, caregivers who can play a vital role in changing attitudes toward tobacco products in patients are found to be the ones with increased consumption of tobacco [12]. Medical professionals are at increased risk of consumption because of their stress or workload, culture, social norms, and values [13]. Tobacco can not only risk the health status of medical professionals; it will also undermine the credibility of the health professional and their ability to deliver effective anti-tobacco counselling programs [14].

Few single-centre studies were conducted in Nepal [15,16]. One study showed a high prevalence (30.1%) [16] of tobacco consumption among medical students. However, there is a lack of nationwide data. Therefore, this study was conducted to assess the prevalence of tobacco consumption, factors associated with tobacco use and to determine the level of nicotine dependence among medical interns in Nepal.

## Materials and methods

### Public and patient involvement

Patients or the public were not actively involved in the design of this study. The medical interns filled out the questionnaire through an online platform. They didn’t have any other role except responding to the questionnaire.

### Study design, participants, and duration of data collection

This analytical, web-based cross-sectional study was conducted among Bachelor of Medicine and Bachelor of Surgery (MBBS) & Bachelor of Dental Surgery (BDS) interns from 22 different medical and dental colleges in Nepal. Those interns (Nepalese) who were doing their internship outside Nepal were excluded from the study, as results may vary due to geographical conditions. Data collection was done from February 15 2023, to April 30, 2023. This study was reported following the Strengthening the Reporting of Observational Studies in Epidemiology (STROBE) guidelines.

Convenience and snowball sampling techniques were used in this study. Initially, a representative intern from each selected college was contacted. These representatives then helped recruit additional participants until the target sample size was reached.

### Ethical approval and informed consent

The study was conducted after receiving ethical approval from the Gandaki Medical College institutional review committee (Reference No:248/079/080). This study follows the guidelines of the Declaration of Helsinki. Written informed consent was obtained from each participant before completing the online questionnaire via a Google survey form.

Participation in the study was voluntary and anonymous. Before accessing the questionnaire, participants were provided with an information statement explaining the purpose of the study, confidentiality measures, and their right to withdraw from participation at any time during completion of the questionnaire by exiting the online survey before submission.

### Sample size calculation

The sample size of this study was calculated using the formula n = [Z^2^P (1-P)/L^2^], where n = required sample size, Z = 1.96 at a 95% confidence level, and P = prevalence of tobacco consumption among medical students in Nepal, which is 30.1% [16]. L = Absolute precision = 5%. The sample size was calculated to be 324. Considering a 20% non-response rate, the calculated sample size was 389. Ultimately, a total of 381 complete and valid questionnaires were successfully collected and analysed. Therefore, the final sample size exceeded the primary statistical requirement of 324 by 57 participants.

### Data collection tool and method

Data was collected through an online survey using a structured questionnaire. The questionnaire and all details were in English because this study group can easily understand English, as their academic language is English. The questionnaire was prepared in a Google Sheet, which consisted of demographic characteristics (Age, sex, residence, stream), tobacco use (yes/no), form of tobacco (smoking/smokeless), age of initiation of tobacco use, and six questions from the Fagerstrom test for nicotine dependence [17] among smokers. The Fagerstrom test for nicotine dependence (FTND) contains six sets of questionnaires, and the total score ranges from 0 to 10.

### Reliability and validity of the FTND Questionnaire

Nicotine dependence was assessed using the Fagerström Test for Nicotine Dependence (FTND), a widely used and validated instrument with established reliability and validity for measuring nicotine dependence.

### Recruitment

Participants were informed about the study’s purpose, with detailed explanations and definitions of key terms provided on the first page of the online survey. Written informed consent was obtained from each participant before completing the questionnaire.

Participants were directed to an anonymous online questionnaire, where they provided written informed consent by ticking (√) “Yes” to the question “Do you agree to participate in this study?” Only one response per participant was allowed. To avoid duplicate submissions, email verification was required.

### Data collection procedure

The questionnaire was sent using different online platforms via email, Facebook Messenger, Viber, WhatsApp, and Instagram. A reminder was sent once a week for five weeks. Those who did not respond to the five reminders were considered non-respondents.

To address the potential for response bias-where non-tobacco users might have been more likely to participate, potentially leading to an underestimation or misrepresentation of tobacco use prevalence-the study assured participants that their responses were anonymous and that no personal information would be disclosed.

There was a total of 26 medical and dental colleges in Nepal. However, during the data collection period, Karnali Academy of Health Sciences (KAHS), National Academy of Medical Sciences (NAMS), and Pokhara Academy of Health Sciences (PAHS) had no medical or dental interns. This was because NAMS and PAHS had not yet begun their MBBS or BDS programs, and KAHS had only recently launched its program. Additionally, no responses were received from the interns of Janaki Medical College. Therefore, this study included responses from interns at 22 medical and dental colleges.

**OPERATIONAL DEFINITION:**

- Current smoker- An adult who has smoked at least 100 cigarettes in his or her lifetime, and who now smokes every day or occasionally in the past 30 days.
- Former smoker- An adult who has smoked at least 100 cigarettes in his or her lifetime but who had quit smoking at the time of the questionnaire fill-up.
- Non-smoker- An adult who has never smoked or smoked less than 100 cigarettes in their lifetime and does not currently smoke [18].
- Smokeless tobacco – Any tobacco product that consists of cut, ground, powdered, or leaf tobacco and that is intended to be placed in the oral or nasal cavity.

Data were collected using the questionnaire provided in Annex I.

### Statistical analysis

Data was imported from Google Sheets and analysed using R software (version 4.4.3). Descriptive statistics, including the mean, frequency, percentage and standard deviations, were computed. The prevalence of tobacco use was calculated as the proportion of participants who reported current use of any tobacco product among all respondents who completed the survey. The chi-square test was used to examine associations between tobacco use and independent variables such as sex, academic stream, and residence. Sex, academic stream and residence were included in a multivariable logistic regression model to identify factors independently associated with tobacco use. Adjusted odds ratios (ORs) with 95% confidence intervals (CIs) were calculated, and a p-value of <0.05 was considered statistically significant. Nicotine dependence was assessed using the Fagerström Test for Nicotine Dependence (FTND), which consists of six items with a total score ranging from 0 to 10. Higher scores indicate greater nicotine dependence. Based on the calculated score for nicotine dependency, dependence was categorised into highly dependent (7–10), moderately dependent (4–6), and minimally dependent (< 4).

The study was originally conceptualised within a descriptive framework; inferential statistics were not pre-specified in the initial study protocol. Instead, exploratory, post-hoc inferential analyses were performed to investigate potential associations between tobacco use and demographic factors. To build the multivariable logistic regression model, we used a forced-entry (“Enter”) method, entering all three demographic variables simultaneously and retaining them in the final model regardless of bivariate significance. No formal adjustment for multiple comparisons was applied because the inferential analyses were exploratory and not prespecified as confirmatory analyses. Given the exploratory nature of the analysis and the limited set of covariates included, the adjusted associations were interpreted cautiously.

## Results

Nationwide, there were approximately 1,560 interns from 22 different medical and dental colleges in Nepal during the study. We distributed the online survey to 798 of them. Of the 798 interns who received the survey, 381 completed it, for a response rate of 47.74%. This represents 24.42% of the total study population. The mean age of the participants was 25.04 ± 1.41 years, ranging from 23 to 31 years.

The majority of participants were male, 207 (54.33%). Almost two-thirds of the participants were from the MBBS stream, 246 (64.57%). The prevalence of tobacco use was found to be 69 (18.11%). Smoking was significantly more common among male and MBBS interns (Table 1).

**Table 1:** Demographic details and tobacco use of the participants (N = 381).

Variables	Non-tobacco users N(%)	Smokers N(%)	Smokeless tobacco users N(%)	p-value
**Sex**				
Male	149 (71.98%)	45 (21.74%)	13 (6.28%)	**< 0.001**
Female	163 (93.68%)	5 (2.87%)	6 (3.45%)	
Total	312 (81.89%)	50 (13.12%)	19 (4.99%)	
**Stream**				
MBBS	193 (78.46%)	40 (16.26%)	13 (5.28%)	**0.04**
BDS	119 (88.15%)	10 (7.41%)	6 (4.44%)	
Total	312 (81.89%)	50 (13.12%)	19 (4.99%)	
**Residence**				
Rent	80 (81.63%)	14 (14.29%)	4 (4.08%)	0.07
Home	131 (80.37%)	27 (16.56%)	5 (3.07%)	
Hostel	101 (84.17%)	9 (7.5%)	10 (8.33%)	
Total	312 (81.89%)	50(13.12%)	19 (4.99%)	

The mean age of initiation of tobacco users was found to be 20.41 ± 2.762 years. Among the tobacco users, 19 (27.54%) responded that their family members also used tobacco, whereas 50 (72.46%) responded that their family members were not using any tobacco products. Almost half of the tobacco users (47.83%, n = 33) regretted the use of tobacco. More than half (56.52%, n = 39) of tobacco users tried to quit at least once in their lifetime. Most of them, 60 (86.96%), consumed alcohol/beer or wine (Table 2).

**Table 2:** Characteristics of tobacco users (N = 69).

Mean age of initiation of tobacco users	20.41 ± 2.762	
Characteristics	Yes	No
Any members of the family using tobacco products	19 (27.54%)	50 (72.46%)
Feeling regret for using any tobacco products	33 (47.83%)	36 (52.17%)
Tried to quit tobacco	39 (56.52%)	29 (42.03%)
Concurrent use of alcohol	60 (87.0%)	9 (13.0%)

Female interns had significantly lower odds of tobacco use compared to male interns (OR = 0.18; 95% CI: 0.09–0.36). No statistically significant association was observed between tobacco use and stream of study or residence status (Table 3).

**Table 3:** Multivariable logistic regression analysis for predictors of tobacco use among medical interns in Nepal (N = 381).

Predictors	OR (95% CI)	p-value
**Sex**		
Male (ref)	1.00	–
Female	0.18 (0.09–0.36)	**<0.001**
**Stream**		
MBBS (ref)	1.00	–
BDS	0.73 (0.38–1.37)	0.339
**Residence**		
Hostel (ref)	1.00	–
Home	1.03 (0.53–2.01)	0.938
Rent	0.84 (0.40–1.78)	0.651

Footnote: OR = Odds Ratio; CI = Confidence Interval, Reference category indicated as (ref)

Among the tobacco users, the most common reason for initiation of tobacco products was self-interest, followed by peer pressure (Table 4).

**Table 4:** Reasons for initiation of tobacco products (N = 69).

Reasons	Frequency N (%)
1. Self-interest	31 (44.93%)
2. Peer pressure	16 (23.19%)
3. Stress factor	10 (14.49%)
4. To socialize	9 (13.04%)
5. Other factors	2 (2.90%)
6. Influence from movies and media	1 (1.45%)

All smokers (n = 50) smoked more than 100 cigarettes in their lifetime and were currently smoking within the last 30 days. However, one of the participants did not fill out the nicotine dependence questionnaire, so a total of 49 were included for nicotine dependence. Among these smokers**,** the nicotine dependence level was minimal in 73.47% (36/49), medium in 24.49% (12/49), and high in 2.04% (1/49).

## Discussion

The overall prevalence of tobacco use in this study was 18.11% (69/381), which is lower than that reported in the general population and slightly lower than the 27% [19] reported in a similar study conducted in India. However, a systematic review and meta-analysis of substance use among medical students in India revealed a tobacco use prevalence of 21.9% [20]. Similarly, national data from Nepal in 2019 indicated that 28.9% of adults aged 15–69 years of age were current tobacco users [11]. The lower prevalence rate in our study may be partly attributed to the younger age group of participants compared to the national sample, as well as potentially greater health awareness among medical interns. Globally, the prevalence of tobacco use showed a declining pattern [21]. This trend partly explains the lower prevalence of tobacco use in this study, particularly as data for the previous study [19] were collected nearly a decade earlier. Another possible reason is the higher educational level of participants in this study. A previous study showed the inverse relationship between education level and smoking rate [22], with individuals having higher educational attainment generally reporting lower rates of smoking**.** A study [23] conducted among medical students in western Nepal reported a slightly lower prevalence of 16.3% compared with this study. The lower prevalence in that study may be attributed to the participants’ younger age (mean 21.36 ± 2.24 years) compared with 25.04 ± 1.41 years in our study.

This study observed that 13.12% of interns used tobacco in the form of smoking, while 4.99% used a smokeless form. The smoking rate was lower among female interns at 2.87% (5/174) than among males at 21.74% (45/207), similar to results reported in other surveys in South Asian countries [24]. Due to conservative societies, social norms, and cultural boundaries, smoking is generally considered unacceptable for females. However, recent surveys show a slight increase in smoking rates among women, likely due to factors such as education, employment, and urbanisation [24]. Thus, the tobacco cessation program should be conducted in both sexes.

The consumption of tobacco products was highest among participants living at home at 19.63% (32/163), followed by those in rented accommodation at 18.37% (18/98) and those residing in the hostel at 15.83% (19/120). These results contradict similar studies in India, which show that the proportion of students in hostels is more prone to consuming tobacco [19,25]. These results are consistent with an away-from-home environment without parental care and peer company. Hence, their place of residence should be the prime target in managing the tobacco consumption rate [26]. The reason for the highest consumption of tobacco at home in this study may be that other family members are also using tobacco in their homes.

This study revealed that the level of dependence was high in 2.04% (1/49), medium in 24.49% (12/49), and minimal in 73.47% (36/49), which signifies very minimal dependence level among medical interns in Nepal as compared to the community-based cross-sectional studies in Nepal where a very high dependence level was found in 16.1% of respondents [27] and a very high dependence level was found in 9% of respondents in a study done in eastern Nepal [28]. This inconsistency may be due to higher levels of education, knowledge, and attitudes toward nicotine-containing substances among medical interns compared to the general community.

However, this finding is noteworthy given that the participants were medical interns who will soon assume roles as healthcare professionals responsible for promoting tobacco cessation and healthy behaviours among patients, including setting examples as non-users of tobacco. Tobacco use and nicotine dependence among healthcare trainees may influence their attitudes toward tobacco control policies and their willingness or confidence to provide cessation counselling.

Among tobacco users, 27.54% (19/69) had a family history of smoking, consistent with a study [29] that demonstrated a link between tobacco use and family patterns. Thus, family history plays an important role in the initiation of tobacco consumption, which should be considered during the design of a tobacco cessation program [26].

Since this study primarily focused on medical interns in Nepal, the findings cannot be generalised to the broader population. Further studies involving a wider and more diverse population are recommended. However, as interns from nearly all medical and dental colleges in Nepal were included despite some uneven representation across institutions these findings provide preliminary insights into tobacco use among medical interns in Nepal; however, due to the sampling strategy, the results should be interpreted cautiously and may not be fully representative of all medical interns nationwide.

### Limitations

Social desirability bias may have led to underreporting of both tobacco use and nicotine dependence levels. Recall bias may have occurred. Psychological stress levels were not assessed in this study, which could have provided deeper insights into the factors influencing tobacco use.

This study utilized convenience and snowball sampling, resulting in a non-response rate of 52.26% (417/798). While low response rates are a systemic challenge in online survey research, where the literature indicates an average response rate of approximately 44.1% [30], the response rate of 47.74% (381/798) sits slightly above this benchmark. Nevertheless, this non-response rate introduces a distinct risk of selection and differential non-response bias. Because data may not have been missing at random for example, if active tobacco users were systematically less likely to participate the validity of the baseline prevalence estimate could be affected. Due to the anonymity of the protocol, characteristics and tobacco-use status of the non-responders were completely unavailable, conducting a formal sensitivity analysis, inverse probability weighting (IPW), or propensity score adjustment was unfeasible. The reported 18.11% prevalence should therefore be interpreted as the rate within this participating sample rather than a definitive representation of all medical interns across Nepal.

Additionally, inferential statistics (chi-square tests and multivariable logistic regression models) were integrated as post-hoc, exploratory analyses rather than a priori pre-specified testing. Because these analyses were strictly exploratory, adjustments for multiple testing were not applied. Consequently, the possibility of Type I errors (false-positive findings) due to multiple testing cannot be excluded. Therefore**,** these secondary findings should be interpreted with caution, with the identified associations intended primarily as hypothesis-generating for future prospective research.”

## Conclusions

Although smoking was prevalent among medical interns, its prevalence was lower than that reported in the general population, and most smokers exhibited relatively low nicotine dependence. Encouragingly, more than half of the participants had made at least one attempt to quit, suggesting a willingness to change. This highlights the potential effectiveness of providing tobacco cessation counselling. Therefore, it is essential to increase awareness and offer cessation support to medical students. If medical interns reduce or completely avoid tobacco use, they will be in a better position to offer effective cessation counselling to tobacco users and inspire others to refrain from using tobacco. Educating medical students about the dangers of tobacco and empowering them to advocate for tobacco prevention can positively impact public health. Additionally, based on these exploratory findings, this study recommends increasing awareness among medical students about the risks of using tobacco alongside other addictive substances, such as alcohol, to discourage concurrent substance use.

## Supporting information

S1 Data De-identified survey responses. This spreadsheet contains the raw dataset used to calculate the prevalence of tobacco use and nicotine dependence among the sampled medical interns in Nepal.
(XLSX)

## Abbreviations

BDS: Bachelor of Dental Surgery
CI: Confidence Interval
FTND: Fagerstrom test for nicotine dependence
KAHS: Karnali Academy of Health Sciences
MBBS: Bachelor of Medicine and Bachelor of Surgery
NAMS: National Academy of Medical Sciences
OR: Odds Ratio
PAHS: Pokhara Academy of Health Sciences
STROBE: Strengthening the Reporting of Observational Studies in Epidemiology
WHO: World Health Organization (WHO)
